# Multi-Modal Contractive Forces of Wools as Actuator

**DOI:** 10.3390/polym12071464

**Published:** 2020-06-30

**Authors:** Shanshan Zhu, Jinlian Hu

**Affiliations:** 1 Institute of Advanced Integration Technology, Shenzhen Institutes of Advanced Technology, Chinese Academy of Sciences, Shenzhen 518055, China; ss.zhu1@siat.ac.cn; 2 Department of Biomedical Engineering, City University of Hong Kong, Kowloon, Hong Kong 999077, China

**Keywords:** wool, wool yarn/polymeric yarn blends, twisted structure, isometric stress, actuator

## Abstract

Wool has a long history of use in textiles throughout human civilization. Many smart functions such as reversible shape changes to various stimuli have been demonstrated in the last few years. However, the force-related characteristics are still imperfectly recognized, although they are expected to be used as actuators due to their biological origins and broad applications. Herein, we investigated the feasibility of wools in performing actuating ability through its intrinsic structures and fabrication methods. The diverse modes of contractive forces were obtained in wool materials including platform-like, double-peak, and slope-like shapes, where a molecular model was also presented to trace the origins of stress evolution. After that, a polymeric blend was created to modify the wool materials and a dissimilar performance of stress production was achieved, a square stress mode with stable manner and maintenance, for broad applications in a more efficient way. It is believed that these actuating properties extracted from natural hairs have a large potential in current smart applications and lay down new inspiration in designing actuators.

## 1. Introduction

Smart textiles are beginning to thrive in the field of artificial intelligence [1,2,3,4,5,6] including actuators [7], energy-harvesting fabrics, energy-storing fabrics [2], flexible sensors with color/temperature/humidity sensing abilities [4,8,9], etc. In particular, characteristics of these textiles like good flexibility, compliance, and light weight have promoted the development of a new generation in biological actuators (e.g., exoskeleton suit [10]). For the application of an actuator in artificial muscles, diverse categories of polymeric fibers through thermal/moisture/electrical management have been investigated on the basis of twisted and coiled structures [2,11], which can yield satisfying torsional and tensile actuation. However, the most widely used materials are not easy to commercialize as wearable materials or smart textiles at present due to the high actuating temperature (e.g., polyethylene) or high price (e.g., carbon nanotubes) [12,13]. Thus, an actuator with satisfying contractive forces, environmentally friendly performance, and low cost is being pursued.

**Scheme 1:**
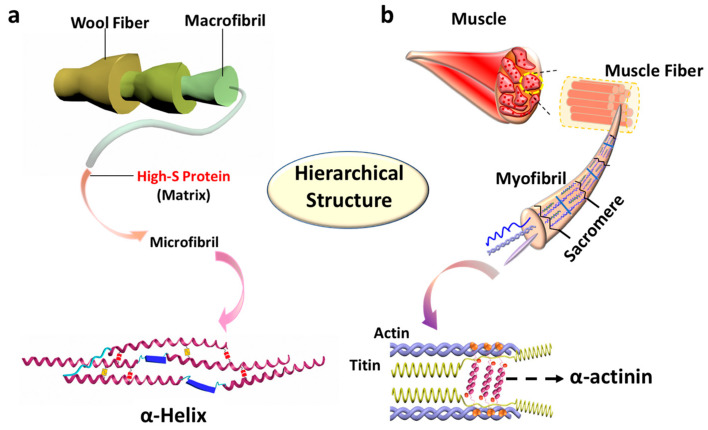
The hierarchical structure of (**a**) wool fiber and (**b**) natural muscle fiber.

Natural hairs have the advantages of low prices, biodegradability, and can be used as environmentally friendly alternatives that are superior to polymeric fibers [14]. Moreover, with the further exploration of wools and camel hairs, the shape memory effect has been discovered and systematically studied as responsive to water, heat, and redox, respectively [15,16]. Among the various stimuli, heating is commonly preferred because the changes of inner components can be easily altered by specific temperature ranges compared to chemical treatments, where more structures would be affected. However, intrinsic characteristics like contractive force for an actuator of natural hairs have rarely been studied. Therefore, the investigations of natural hairs on structure and properties, especially the stimulus–responsive force for actuating applications, have aroused our interest. Based on previous studies, wool has a heterogeneous composition and a complex hierarchical structure (Scheme 1a). Alpha-helix, one of the most important secondary structures of proteins, involves both hydrogen bonds (HBs) and disulfide bonds (DBs) inside. In terms of structure, some similarities have been found between animal hairs and skeletal muscle (Scheme 1b) [17] including the hierarchical structure, the existence of a dynamic bond, crystal-like structure, and the trainable component like actin in muscle, which have provided a lot of inspiration to well utilize the natural ones. In the sarcomere structure of muscle, which has emerged as a kind of multipurpose protein, α-actinin can not only interact with actin filaments, but can also participate in contractile machinery, regulating structures and responding to signaling proteins [18]. It was also found that α-actinin could crystallize [19] and possesses twisted antiparallel structures [18]. Based on these similarities between the two structures of wool and muscle fiber [20], we assumed that wools and wool yarns could also acquire analogous actuating abilities, which could be further applied to artificial muscle.

Notwithstanding the advantages of animal hairs, there are still concerns. The low fatigue resistance of wools caused by weak elasticity [21] will produce a large energy dissipation in later cycling. Our previous study [22] designed and prepared a kind of smart polymeric material with low energy dissipation and good thermal-contraction ability, which could be taken advantage of to design a wool yarn/polymeric yarn blend to make up for the deficiency. In this study, the thermomechanical properties including isometric contraction and isothermal tensile hysteresis cycles of raw wools, wool yarns, and wool yarn/polymeric yarn blends were investigated, together with various fabricated structures. Then, the corresponding inner structure changes covering the degree of crystallization and bond changes were characterized using Fourier transform infrared (FTIR), x-ray diffraction (XRD), and differential scanning calorimetry (DSC). It was proven that the multi-modal isometric stress could be realized, and wool yarn/polymeric yarn blend could be used to further improve the utility.

## 2. Materials and Methods

The raw hair fibers used in this study was from a goat’s back, purchased from a trade factory (Sunite Right HTC villi LLC, Mongolia Autonomous, China). The selected hair fibers all had a diameter of more than 50 µm. Two-ply wool yarn was purchased from the same company as the raw wool fibers. Stress–memory polymeric fibers was in-house synthesized by Polycaprolactone (PCL, Mn = 4000 g mol^−1^) forming a soft segment and methylene diphenyl diisocyanate (MDI) with its extender butanediol (BDO) forming a hard segment [22]. The sample was dissolved in dimethylacetamide (DMAC) and subsequently spun into fibers. The twisted structure of the raw wools was produced by twisting a bundle of wool fibers to near, but not reaching the point of coiling. Four-ply wool yarn was produced by twisting two of the purchased two-ply wool yarns. The four-ply wool yarn/polymeric yarn blend was produced by twisting a purchased two-ply wool yarn and a two-ply polymeric yarn together to near, but not reaching the point of coiling.

FTIR analysis was carried out on a PerkinElmer spectrum 100 FTIR spectrometer (Suzhou, China) with a frequency range from 4000 cm^−1^ to 650 cm^−1^. Each spectrum was obtained by averaging 16 scans with a 4 cm^−1^ resolution. These spectra were corrected by advance attenuated total reflection (ATR) correction before quantitative analysis. The molecular structures and bond stretching of wool and yarn were studied by FTIR. The transition temperatures (T_trans_) and enthalpy change of both the raw and programmed specimens were determined by differential scanning calorimetry (DSC) using a Perkin–Elmer Diamond instrument with nitrogen as protection (Shanghai, China). The specimen was first kept at 20 °C for 1 min to balance the environment and then a heating scan was conducted from 20 °C to 250 °C with a heating rate of 10 °C/min. A Rigaku SmartLab 9 kW—Advance XRD (Oakland, CA, USA) machine was used to characterize the crystallinity of the raw wool, wool yarn, and stretched wool yarn, where the scanning intensity was recorded in the range of 5–40°. Finally, an Instron 5566 tension machine (Hong Kong, China) equipped with a temperature-controlled oven was used to explore the mechanical properties, isometric contraction, and hysteresis of the specimens.

## 3. Results and Discussion

### 3.1. Platform-Like and Double-Peak Stress-Stroke Mode of Raw Wools

**Figure 1:**
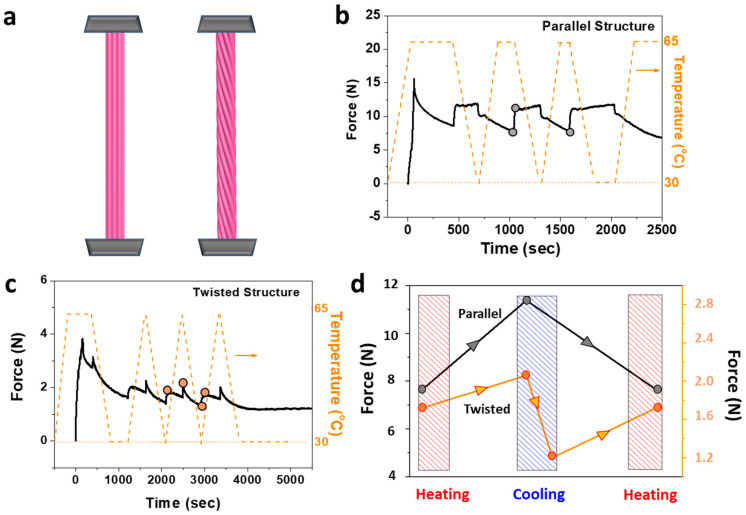
(**a**) Raw wool fibers in the parallel and twisted structures. Isometric contraction test of wool fibers in (**b**) parallel and (**c**) twisted structure responding to the stimuli of temperature. (**d**) Evaluation of force change in two structures: adopted data were acquired from the marked points as shown in (**b**) grey circles and (**c**) orange circles.

After stretching the samples to the strain of 10%, the contractive force in the strain constraint condition responding to thermal changes on raw wools in the form of both parallel and twisted structures were investigated first, as shown in Figure 1a. Wools with a parallel structure (Figure 1b) were first heated to 65 °C (T_high_) and stretched to the strain of 10%, where the force increased to about 15 N. Then, a reversible thermal protocol was applied between room temperature (RT) and T_high_, where the isometric force increases in cooling due to contraction, stabilizes at a platform due to a stable structure at RT, and decreases immediately in heating due to the fast stress and structure relaxation [23] at T_high_. However, it is interesting that a totally contrary effect was found for raw wools in the twisted structure, as illustrated in Figure 1c, where the evolution of isometric force after stretching was similar to that of stress–memory polymers (SMPs) in our previous work [22]. First, a large stress drop appeared in cooling after tension at T_high_, following a sharp stress–stroke at the initial moment. Then, a smooth stress–stroke was produced in heating, followed by a gradual stress decrease. The characteristic smooth stress–stroke and sharp stress–stroke could be cyclically realized by further changing the thermal stimulus. Figure 1d plots the isometric force dependence of temperature for wools in two structures, where the parallel structure exhibited a larger tension force and output force due to high stiffness. The force stroke was produced only in cooling for the parallel structure, but a double-peak stroke could be obtained in both heating and cooling for the twisted one. Furthermore, unlike the downward trend of overall forces in the twisted structure after several cycles due to the cost of stiffness, the overall force in the parallel structure could be better maintained to a large proportion and presented a platform-like mode.

**Figure 2:**
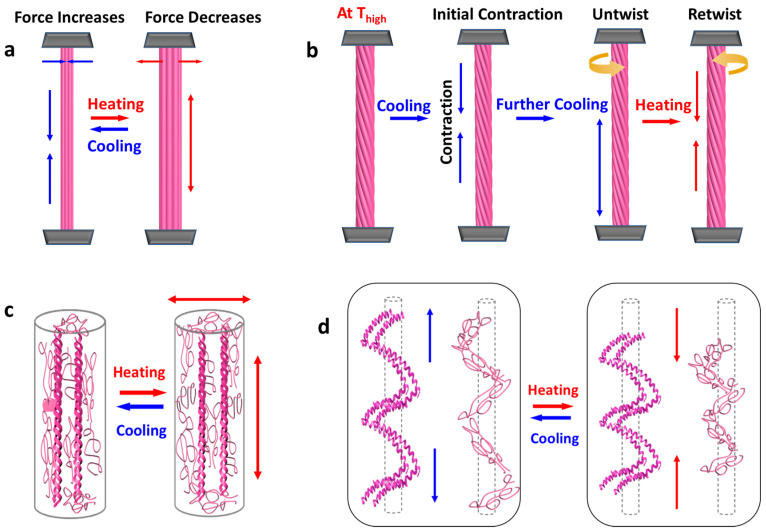
(**a**) Thermal expansion of raw wools in the parallel structure. (**b**) Untwisting and retwisting manner of wools in the twisted structure. (**c**) Cylindrical two-phase model of anα-keratin fibers in thermal conditions. (**d**) Conformational entropy changes of both alpha-helices and the amorphous matrix in the twisted structure.

Unlike the thermal–contraction character of fibers like nylon [12], wool fibers exhibit a positive axial thermal expansion. In order to analyze the different origins of force actuation in two structures, a two-phase model of wool fiber was used for the parallel structure, as presented in Figure 2a,c, where the fiber was composed of an amorphous matrix and embedded alpha-helices [23]. Upon heating, the polymer chains in amorphous regions absorb heat and acquire decreased moduli as well as isotropical expansion, directly resulting the stress drop in the constrained condition. In addition, it is speculated that the exchange of disulfide bonds (DBs) inside occurs at the selected temperature T_high_ [24], facilitating the increase in the length of the sample because there is less restriction for the thermal expansion of the matrix [25]. For the twisted raw wools, as depicted in Figure 2b,d, the sharp stress–stroke occurred at the beginning of cooling due to the instant increase in modulus from the intrinsic properties of the fibers, while the further stress drop resulted from the untwisting manner (Figure 2b). Once the heating started again, the contractive force yielded due to retwisting, where the double-peak stress–stroke could be achieved. The major contribution to this negative thermal expansion is presumed to come from the amorphous matrix and alpha-helices along the path of the macroscopic twist (Figure 2d). It was discovered that the tension force for wools in the twisted structure was much lower than that of the parallel one, indicating that the stiffness of the sample could be effectively decreased through twisting, hence allowing more conformational freedom [26]. As a result, the conformational entropy change will occur during heating like rubber elasticity and then the contractive force can be produced.

### 3.2. Slope-Like and Double-Peak Stress–Stroke Mode of Plied Wool Yarns

**Figure 3:**
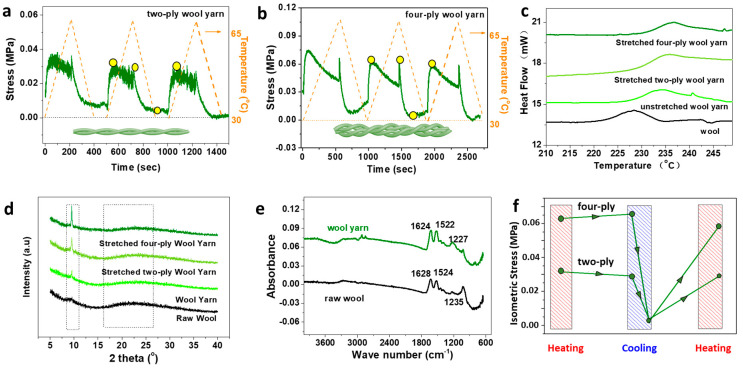
Isometric contraction of (**a**) two-ply wool yarn and (**b**) four-ply wool yarn. (**c**) Differential scanning calorimetry (DSC), (**d**) X-ray diffraction (XRD) pattern and (**e**) Fourier transform infrared (FTIR) of samples with and without stretching. (**f**) Evaluation of stress evolution: adopted data were acquired from marked points as shown in Figure 3a,b (yellow circles).

Then, the isometric contraction of plied wool yarns, as illustrated in Figure 3a,b, showed that the stress evolution with temperature had a similar pattern with that of the twisted raw wools. The slope-like and double-peak stress–stroke could be found in the two-ply and four-ply wool yarns, respectively. It is also applicable to wool yarn that with an increase in the mechanical cycle, the rigidity of the keratin structure was reduced, leading to a sustained downward trend for stress values. Then, the characterization of structures of both unstretched and stretched samples was performed as shown in Figure 3c–e to explore the various thermomechanical manners in contraction. An endothermal peak was observed for all samples in the DSC curves (see also Figure 3c) when the temperature was higher than 220 °C that should be ascribed to the denaturation of the helical protein in the fibers [27]. For the original wool fiber, the intensity of the peak was located at 228 °C while the position of the peak increased in the plied wool yarns and became higher in the stretched wool yarn. The increased temperature for the crystal cleavages in wool yarns may perhaps be due to the better ordered structures obtained in yarns compared to the raw wools. Moreover, the increased enthalpy changes as well as transition temperature indicate the tension effect on crystallization. X-ray diffraction patterns in Figure 3d also support this statement. The diffraction peak at about 9.45° was weak in the sample of raw wool fibers, but became a little bit stronger in the wool yarns. With stretching, the mobility of the molecular chain segments will change, further influencing the alpha-helices in the fiber. The increase of crystallinity might indicate the transfer from the alpha-keratin structure to the beta-keratin structure with stretching [28]. The secondary structure of proteins were obtained through FTIR spectroscopy [29], where peaks at about 1624 cm^−1^ and 1522 cm^−1^ of wool yarn and 1628 cm^−1^ and 1524 cm^−1^ in Figure 3e are attributed to the stretching vibration absorption of the free carbonyl group (C=O) and hydrogen bonded carbonyl group (C=O), respectively. In common, hydrogen bonded C=O was located at low frequency ranges compared to the free one [30]. The absorbency peak at 1624 and 1628 cm^−1^ should be assigned to the elastic vibration of the C=O bond, which is commonly observed for the beta-form crystal [25]. The regularity of the molecular structure in stretched wool yarn is greater than that in the original one, inducing better crystal structure or even transformation from the alpha-form to beta-form. During this process, the characteristic peaks above-mentioned shifted two lower wave-numbers in the wool yarn, corresponding to better crystal structure compared to the raw wools [31]. Figure 3f evaluates the isometric stress change responding to temperature. Improved actuating performances from four-ply wool yarn should be attributed to larger tension stress, but it does not exclude the possibility of enthalpy change from increased crystallinity, which can be partly melted at 65 °C and facilitate the stress production [32].

### 3.3. Stepwise and Square Stress–Stroke Mode

**Figure 4:**
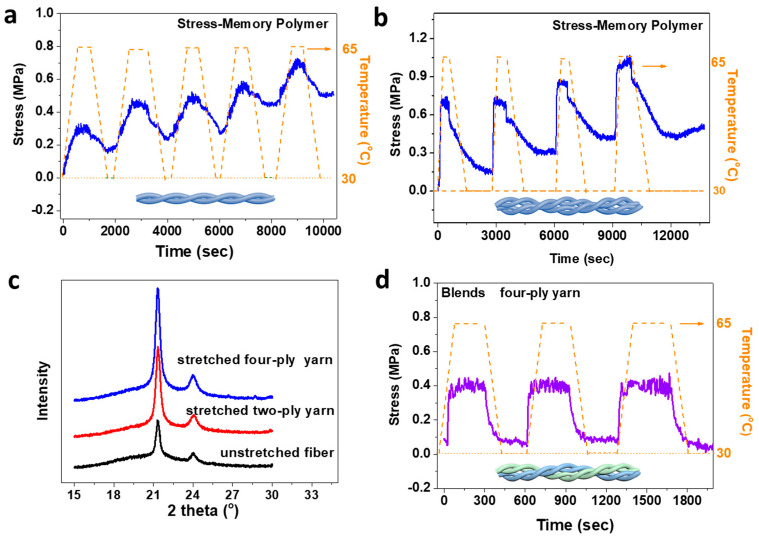
Isometric contraction of (**a**,**b**) two-ply and four-ply stress–memory polymeric yarns. (**c**) XRD characterization of stress–memory polymer fiber and yarns. (**d**) Isometric contraction of four-ply wool yarn/polymeric yarn blends.

For stress–memory polymeric yarn, the isometric stress actuation in heating is caused by the enthalpy change of semicrystals during transition, inducing the energy to be released in the form of stress [33]. Unlike the stress evolution of contraction in the form of film [22], the stress value increases continually with actuation cycles in a stepwise manner (Figure 4a,b), which is induced by the better-formed orientation as well as crystallinity in polymeric fibers (Figure 4c) [34]. As discovered, the raw wools with a twisted structure could produce stress–stroke to temperature, where the development of stress had the same direction with that of the SMP. The isometric stress versus temperature for samples with twisted structure of blended wool yarn/SMP yarn are characterized in Figure 4d. As expected, the downtrend stress in wool yarn could be neutralized by the upward stress evolution of SMP yarn. Then, a kind of square stress–stroke was acquired with a relatively stable and controlled manner. With regard to pure wool plied yarns, polymeric fibers not only provide structural reinforcement, but can also erase the stress relaxation in an actuated state. In the blends, the isometric stress produced was associated with both semi-crystal-melt transition and thermal contraction resulting from both the micro- and macro-structures.

**Figure 5:**
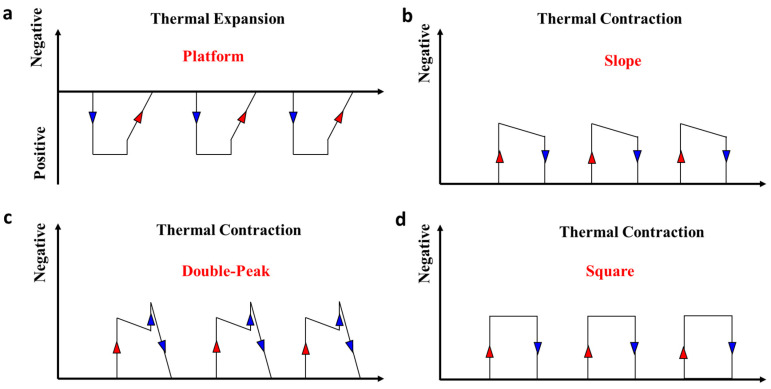
Multi-modal contractive forces of the wool, wool yarn, and wool yarn/polymeric yarn blends. (**a**) Platform-like stress–stroke mode originated from raw wools in the parallel structure. (**b**) Slope-like stress–stroke mode originated from two-ply wool yarn. (**c**) Double-peak stress–stroke mode originated from raw wools in the twisted structure and four-ply wool yarns. (**d**) Square stress–stroke mode originated from four-ply wool yarn/polymeric yarn blends.

The multi-modal contractive forces of the tested specimens are summarized in Figure 5. As illustrated in Figure 5a, the platform-like stroke in wools with a parallel structure resulted from the positive thermal expansion as explained in Figure 2c. The slope-like stroke of two-ply wool yarn in Figure 5b came from both the micro- and macroscopic entropy change (negative thermal expansion), where both the amorphous matrix and twisted structure tended to contract in heating. The double-peak stroke in raw wools with the twisted structure and four-ply wool yarn is illustrated in Figure 5c, which was similar to the stress evolution of the slope-like one. The only difference was discovered at the beginning of cooling, where a sharp stress increase appeared, which resulted from the positive thermal expansion. This is because the specimen with the four-ply structures had a higher modulus compared to the two-ply one, then the cooling-induced modulus increase produced a larger influence on stress at that time. Finally, the square stress in Figure 5d came from the four-ply wool yarn/polymeric yarn blend, where a stable stress stroke was obtained due to the combination of the enthalpy change of SMPs, thermal expansion, and the conformational entropy change of the twisted wool yarns.

### 3.4. Tensile Hysteresis

**Figure 6:**
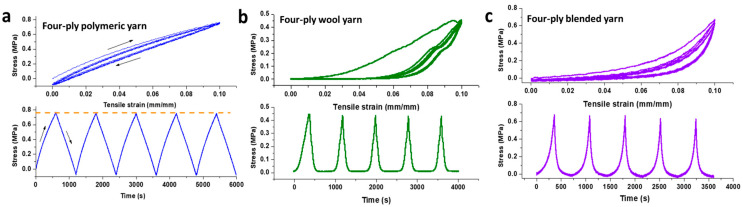
Isothermal tensile hysteresis cycles at T_high_ of (**a**) stress–memory polymeric yarn, (**b**) wool yarn, and (**c**) four-ply wool yarn/polymeric yarn blends.

Energy dissipation is generally evaluated through the tensile hysteresis curve, where differences of stress–strain curves among different cycles could directly reflect the change of inner/outer structure of materials. Here, the isothermal tensile hysteresis tests were performed at T_high_ for SMP, the wool yarns, and blends, as illustrated in Figure 6. First, heating and keeping the sample at T_high_ for at least 30 min to erase the thermal history, then stretching the sample to the strain of 10%. Subsequently, clamps on both heads of the sample returned to zero tension strain with the rate of 1 mm/min and repeated the procedure for five cycles. As illustrated in Figure 6a, the tension and recovery curves of SMP almost overlapped with each other from the second cycle, reflecting that a stable elastic network was produced. Furthermore, the energy dissipation from the first to second cycle was not that much. However, a high hysteresis loss was found in the pure wool yarns as presented in Figure 6b, where the stress–time curve was not as symmetrical as that of the SMP. This showed that the structure changed a lot from the second cycle and the major reason is the breakage of the inner/outer structure during tension. As with the result of Figure 4d, the better performance was achieved in blends compared to that of wool yarns, where the stress–strain curve could be smooth (Figure 6c), and less energy dissipation from the first to second cycle was realized.

## 4. Conclusions

As a featured natural hair, wools display more possibilities in the application of smart textiles, where the existence of isometric stress and potential of an actuator were proven. Compared to the normal thermal expansion for raw wools in the parallel structure, a double-peak stress–stroke mode (one smooth and one sharp manner) was discovered in the thermal stimulation in the twisted structure as well as in the wool yarns. Both the positive thermal expansion from the molecular motion of the amorphous phases and the negative thermal expansion from the untwisting and retwisting were attributed to the development of contractive stress. Then, the combination of stress memory polymeric yarn and wool yarn gave the blends a square stress-mode with alleviative structure relaxation and less tensile hysteresis, which covered the shortage of high energy dissipation in pure wool materials. We envision that these actuating properties extracted from nature and the proposed blends will provide valuable insights into the development of excavating stress-based smart natural materials with multi stress–stroke modes.
